# Oxytocin—not always a moral molecule

**DOI:** 10.3389/fnhum.2013.00010

**Published:** 2013-01-30

**Authors:** Ulrich J. Pfeiffer

**Affiliations:** Department of Psychiatry, University Hospital of CologneCologne, Germany

**A commentary on**

**The other side of the coin: oxytocin decreases the adherence to fairness norms**

by Radke, S., and De Bruijn, E. R. A. (2012). Front. Hum. Neurosci. 6: 193. doi: 10.3389/fnhum.2012.00193

Oxytocin is a small neuropeptide that has long been implicated in mating and reproductive functions. In recent years, however, it has received increasing attention for its involvement in the regulation of social cognition. A study by Ernst Fehr and colleagues (Kosfeld et al., 2005), in which the intranasal administration of oxytocin increased the trusting behavior of investors toward strangers in an economic trust game, triggered a growing body of studies demonstrating a plethora of positive effects of oxytocin on social behaviors. It was found to enhance mentalizing abilities, empathy, generosity, prosocial behavior, and to improve the recognition of emotional facial expressions as well as their affective evaluation (Macdonald and Macdonald, 2010). The discovery of these prosocial effects has created an immense media hype during the last years which has taken absurd dimensions with oxytocin being called the hugging hormone, cuddle chemical, a moral molecule, and worse. The gist of the media coverage of oxytocin is that the molecular substrate of good and evil has finally been discovered and that a sniff of this substance might be sufficient to restore love, understanding, and peace in the world. In light of such oversimplifications, attention needs to be directed to the complexity of its function, which is both state- and trait-dependent and thus linked to a variety of complex biological and contextual influences (Churchland and Winkielman, 2012).

An interesting and elegant study in this journal addressed how oxytocin modifies the adherence to fairness norms when subjects played an ultimatum (UG) and a dictator game (DG) with anonymous interactors (Radke and De Bruijn, 2012). In the UG, two players decide how to split an amount of money between them. The proposer offers a certain split and the recipient can either accept or reject her proposal. In the latter case, neither of the players receives anything. In the DG, the proposer is endowed with an amount of money which she can share with a recipient. In this case, the recipient has to accept the proposer's offer. A previous report demonstrated that the administration of oxytocin results in an increase of generosity by proposers in an UG which was attributed to an increase of empathy (Zak et al., 2007). As *proposer* behavior in the UG has been related to strategic considerations (Prasnikar and Roth, 1992), Radke and De Bruijn explicitly sought to investigate *responder* behavior which is more directly associated with fairness. To emphasize the focus on fairness, they used a modified, explicit version of the UG in which proposers had to split 10 coins according to one of two alternatives: An unfair offer (8/2) was either paired with a fair alternative (5/5) or with a no-alternative (8/2). Initially, subjects received an intranasal dose of 24 IU of oxytocin. After multiple UG trials in which subjects allegedly saw the proposals of previous proposers, they played a single-shot DG in order to obtain a measure of unconditional altruism not influenced by strategic deliberations. Results showed that rejection rates for unfair offers were higher in the presence of a fair alternative, which is consistent with the results of previous work using the modified UG (e.g., Falk et al., 2003). Intriguingly, however, in comparison to the placebo condition oxytocin did not universally decrease or increase rejection rates, but reduced the effect of the context of an offer: Rejection rates for unfair offers were increased when no fair alternative was present but reduced in the presence of a fair alternative. Furthermore, subjects made more unfair offers after oxytocin administration in the DG with about 60% offering zero coins to the responder as compared to 30% in the placebo condition.

In sum, these findings demonstrate that the fairness context of an offer is discounted and that unconditional generosity is decreased, thereby suggesting that oxytocin diminishes fairness in anonymous games. This is consistent with recent work suggesting that the prosocial effects of oxytocin are parochial, that is, they are narrow in scope and only affect members of the in-group. For example, oxytocin leads to in-group favoritism and results in non-cooperative, defective and even aggressive behavior toward out-group members (De Dreu, 2012). It can be assumed that in experimental settings commonly used to study social behavior, minimal familiarity of interactors and brief reciprocal interactions are sufficient to create an in-group. In Radke and De Bruijn's study, subjects neither had any information about the other nor were able to interact with them. Such an anonymous context might favor the classification of interaction partners as out-group members, especially because oxytocin has been argued to enhance social categorization. The effect of oxytocin hence might be twofold in that it first supports the categorization of an entirely anonymous interaction partner as out-group which subsequently leads to a decrease in the adherence to fairness norms during the UG and the DG. From a more folk psychological perspective these findings aptly exemplify the idiom “better the devil you know than the devil you don't” which exists across many cultures and emphasizes the aversive quality of dealing with uncertainty in social encounters. Indeed, the risk of non-reciprocation is high when dealing with a completely unknown person. In evolutionary terms, it might therefore be beneficial not to cooperate. Interestingly, it has also been suggested that social approach behaviors do not necessarily need to have positive emotional valence (Kemp and Guastella, 2011). For instance, anger or fear can induce aggressive behavior related to altruistic punishment or protecting the offspring. Despite their negative valence, such behaviors are hence counted among social approach behaviors and have been reported to be similarly enhanced by oxytocin as prosocial behaviors. These and other recent results demonstrate that the oversimplified view of the “moral molecule” is misleading and treacherous. Although further research is needed, it is conceivable that depending on the interaction partner and the context of an interaction, oxytocin might exert as many antisocial (or negative approach-related) as prosocial effects.
